# Low-intensity pulsed ultrasound reduces lymphedema by regulating macrophage polarization and enhancing microcirculation

**DOI:** 10.3389/fbioe.2023.1173169

**Published:** 2023-05-05

**Authors:** Zihao Liu, Jia Li, Yu Bian, Xiaojie Zhang, Xiaojun Cai, Yuanyi Zheng

**Affiliations:** ^1^ Department of Ultrasound in Medicine, Shanghai Jiao Tong University School of Medicine Affiliated Sixth People’s Hospital, Shanghai, China; ^2^ Department of Neurology, Shanghai Jiao Tong University School of Medicine Affiliated Sixth People’s Hospital, Shanghai, China

**Keywords:** macrophage polarization, low-intensity pulsed ultrasound, circulation, lymphedema, inflammation

## Abstract

**Background:** Conventional therapies reduce lymphedema but do not cure it because they cannot modulate the pathophysiology of secondary lymphedema. Lymphedema is characterized by inflammation. We hypothesized that low-intensity pulsed ultrasound (LIPUS) treatment could reduce lymphedema by enhancing anti-inflammatory macrophage polarization and microcirculation.

**Methods:** The rat tail secondary lymphedema model was established through the surgical ligation of lymphatic vessels. The rats were randomly divided into the normal, lymphedema, and LIPUS treatment groups. The LIPUS treatment (3 min daily) was applied 3 days after establishing the model. The total treatment period was 28 days. Swelling, fibro adipose deposition, and inflammation of the rat tail were evaluated by HE staining and Masson’s staining. The photoacoustic imaging system and laser Doppler flowmetry were used to monitor microcirculation changes in rat tails after LIPUS treatment. The cell inflammation model was activated with lipopolysaccharides. Flow cytometry and fluorescence staining were used to observe the dynamic process of macrophage polarization.

**Results:** After 28 days of treatment, compared with the lymphedema group, the tail circumference and subcutaneous tissue thickness of rats in the LIPUS group were decreased by 30%, the proportion of collagen fibers and the lymphatic vessel cross-sectional area was decreased, and tail blood flow was increased significantly. Cellular experiments revealed a decrease in CD86^+^ macrophages (M1) after LIPUS treatment.

**Conclusion:** The transition of M1 macrophage and the promotion of microcirculation could be responsible for the beneficial effect of LIPUS on lymphedema.

## 1 Introduction

Lymphedema is a progressive disease caused by lymphatic transport dysfunction primarily because of external or congenital abnormalities. At the late stage of lymphedema, patients experience tissue fibrosis, fat deposition and inflammation, decreased quality of life (QOL), and recurrent infections. Secondary lymphedema is a common complication of cancer treatment (Rockson et al., 2019). Recently, with the advancement of cancer treatment technology, the life expectancy of cancer survivors has increased, leading to lifelong lymphedema in approximately one-fifth of patients undergoing cancer treatment (Fish et al., 2020). Lymphedema patients experience associated symptoms such as chronic pain, dysfunction, repeated skin infections, poor body shape, depression, and anxiety, which can seriously affect their quality of life and mental health (Ahmed et al., 2008). Lymphedema imposes a substantial biomedical burden; therefore, it is crucial to investigate effective treatment options.

The primary therapeutic objective of secondary lymphedema is to improve the patient’s quality of life. Lymphedema treatment can be divided into surgical and conservative treatments (Schaverien and Coroneos, 2019). Surgical treatment methods such as venous lymphatic anastomosis have demonstrated some promise. However, surgical injury and the risk of infection reduce patient acceptance. The objective of conservative treatments, such as manual lymphatic drainage and skin care, is to reduce the accumulation of lymph in the tissues through various measures, reduce edema, and prevent disease progression. Conservative treatments are reportedly not able to have a therapeutic effect on lymphatic structures (Devoogdt et al., 2023). These treatments cannot effectively treat lymphedema, and the effect was unsatisfactory (Sanal-Toprak et al., 2019; Liang et al., 2020). Because of the complexity of current treatment methods and the need for lifelong treatment, lymphedema patients often experience poor compliance. These factors contributed to the patients’ poor QOL (Farncombe et al., 1994). After recovering from cancer surgery, secondary lymphedema patients urgently need a convenient and effective treatment method that can improve their QOL.

Studies have demonstrated that inflammation is a key component of the pathophysiology of lymphedema. Lymphatic obstruction and lymph siltation can continuously irritate the edematous site, resulting in chronic inflammation and exacerbating lymphedema (Grada and Phillips, 2017). When lymphedema occurs, lymph stasis leads to lymphangitis, accompanied by upregulation of inflammatory factors (Yuan et al., 2019). Because of the stimulation of the inflammatory environment, macrophages infiltrate the fusion sites and clear tissue fragments. Traditionally, macrophages are divided into two subgroups: M1 macrophages and M2 macrophages (Trus et al., 2020). Activated M1 macrophages engulf and destroy microorganisms, which is essential for pathogen resistance, but M1 macrophages simultaneously release high levels of proinflammatory factors, such as TNF- α, Interleukin 6 (IL-6), and IL-1 β (Viola et al., 2019).

The expression of inflammatory factors increased at the site of lymph node obstruction, macrophages were polarized by the stimulation of the inflammatory microenvironment, and there was a high expression of polarized M1 macrophages (Liu et al., 2014). The M1 macrophages aggravate the expression of inflammation response. Severe inflammatory responses lead to more severe tissue damage and fibrosis. (Yunna et al., 2020).

Ultrasonic therapy—a form of physical therapy—has made significant progress in recent years because of its unique non-invasive treatment and user-friendly application (Yang et al., 2019). Ultrasound treatment has numerous effects (Jiang et al., 2019). Through the vibration, loosening, and shock of the lesion, ultrasonic therapy can cause cell and tissue movement, produce mechanical effect through internal massage, promote metabolism, strengthen circulation (Yang et al., 2019; Joiner et al., 2022), improve tissue nutrition, and alleviate body inflammation (Ling et al., 2017).

LIPUS can potentially reduce tissue inflammation by regulating macrophage polarization and reducing M1 polarization (Zhang et al., 2019). Theoretically, the mechanical stress of LIPUS may reduce the inflammatory response after lymphedema treatment.

The objective of our study was to provide an experimental basis for the application of LIPUS treatment to patients with secondary lymphedema and then evaluate the microcirculation and macrophage inflammation.

## 2 Materials and methods

### 2.1 Animals

A total of 18 male SD rats (age: 6 weeks; weight: 220–250 g) were obtained from the Animal Laboratory of Shanghai Jiao Tong University Affiliated Sixth People’s Hospital. All animals were housed at 22°C room temperature with a 24-h light/dark cycle. Animal operating protocols were followed in accordance with the laboratory of Shanghai Jiao Tong University Affiliated Sixth People’s Hospital (No. DWLL 2022–0601).

### 2.2 Rat model of tail lymphedema

The rats were randomly divided into three groups (*n* = 6): the normal group, the lymphedema group, and the LIPUS treatment group. To construct a rat tail lymphedema model, we anesthetized the rats with pentobarbital sodium (50 mg/kg intraperitoneal injection). To demonstrate the lymphatic network, we injected 0.1 mL of 2% methylene blue solution intradermally into the end of the rat tail. After disinfecting the skin, a 1 cm circular incision was made at 13 cm–14 cm from the end of the tail. After removing the skin and subcutaneous tissue, the dermis and superficial lymphatic network were eliminated. The collecting lymphatics under the deep fascia on both sides were eliminated. The muscles, tendons, bones, and main subdermal vessels were not injured (Jin et al., 2021).

### 2.3 LIPUS treatment of lymphedema

**FIGURE 1:**
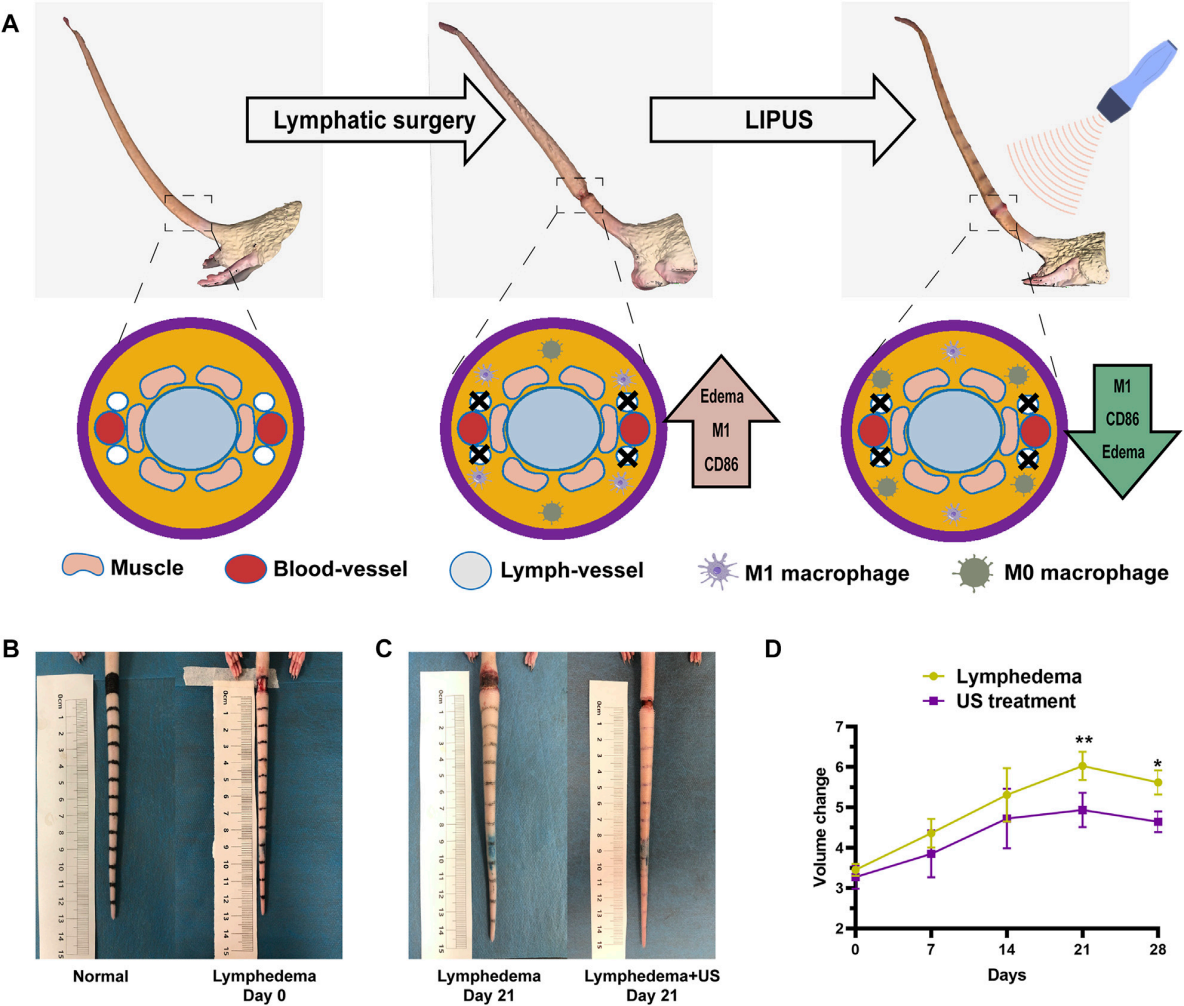
The tail volume of the ultrasonic treatment group and the non-treatment group. **(A)** 3D simulation of acquired lymphedema surgery. The lymphatic network was eliminated. **(B)** Bright-field images of the acquired lymphedema surgery. **(C)** Images of the control group and LIPUS group on day 21. **(D)** The lymphedema tail volume responses of the LIPUS treatment group and the non-treatment group after a treatment course of 28 days. The volume was measured by the drainage method.

The treatment group received ultrasound therapy with the WED-100 all-digital ultrasound therapy instrument (Shenzhen Welld Medical Electronics Co., Ltd.). During ultrasound treatment, the probe was centered on the surgical stump of the lymphatic vessel in the rat tail. Each ultrasonic treatment was administered daily for 3 min (Figure 1A).

LIPUS device parameters: Effective ultrasonic intensity of 0.5 mW/cm^2^; working ultrasonic frequency of 1.0 MHz, 10 ms pulse repetition period; the therapeutic probe is 2 cm^2^. During LIPUS treatment, the rats were anesthetized with isoflurane. The LIPUS probe was carefully fixed on the tail’s skin. The coupling gel was applied to ensure that the sensors were in contact.

### 2.4 ELISA

Excessive pentobarbital sodium is used to euthanize rats and the edema tissue (the skin and subcutaneous tissue) of tail at 13 cm–14 cm was collected. The serum level of VEGF-C were measured using the appropriate kit at day 21. The serum level of IL-1, TNF-α were measured 3 days after LIPUS treatment. All methods were performed according to the manufacturer’s protocol.

### 2.5 Immunohistochemistry and histology

The skin tissue of the rat tail at the surgical incision was sectioned on day 28 post-surgery, and HE staining was used to observe the thickness of the skin and the subcutaneous tissue. Masson’s staining was used to evaluate the formation of collagen fibers.

Anti-Lyve-1 (ab36993) was used to stain LECs. Nuclei were stained with DAPI (Vector Laboratories). All images were measured using ImageJ (National Institutes of Health, NIH).

### 2.6 Cells LIPUS treatment

The raw264.7 cells were seeded in 6-well plates, cultured for 1 day, and then the medium was replaced with one containing lipopolysaccharides (LPS). Ultrasound treatment was initiated after the medium replacement. LIPUS device parameters: Effective ultrasonic intensity of 0.3–0.5 mW/cm^2^, working ultrasonic frequency of 1.0 MHz, and treatment duration of 3 min daily.

### 2.7 Flow cytometry

For macrophage polarization analysis, the raw264.7 cells were labeled with FITC-conjugated anti-CD86 [BU63] (ab77276, Abcam). Flow cytometry data were analyzed and presented using the FlowJo software (Flowjo LLC, Ashland, OR, United States).

### 2.8 Microcirculatory assessment

The tail blood flow of rats in the treatment groups was evaluated using laser Doppler blood flow imaging and a photoacoustic imaging system. Infrared images were used to monitor temperature fluctuations.

### 2.9 Statistics analysis

All data were statistically analyzed with GraphPad Prism 8 (GraphPad, Chicago, IL, United States). The data are expressed as means ± SD or SEM for continuous variables. For comparing the values of the three groups, the one-way ANOVA method was used. A *p*-value of <0.05 was considered statistically significant.

### 2.10 Study approval

The rats in each group were anesthetized so that the tissues in their tails could be sampled for all experiments. This study was approved by the Animal Welfare Ethics Committee of Shanghai Sixth People’s Hospital. All methods were conducted according to relevant guidelines and regulations.

## 3 Results

### 3.1 Low-intensity pulsed ultrasound (LIPUS) reduced the edema and fibrosis of rat tail lymphedema

**FIGURE 2:**
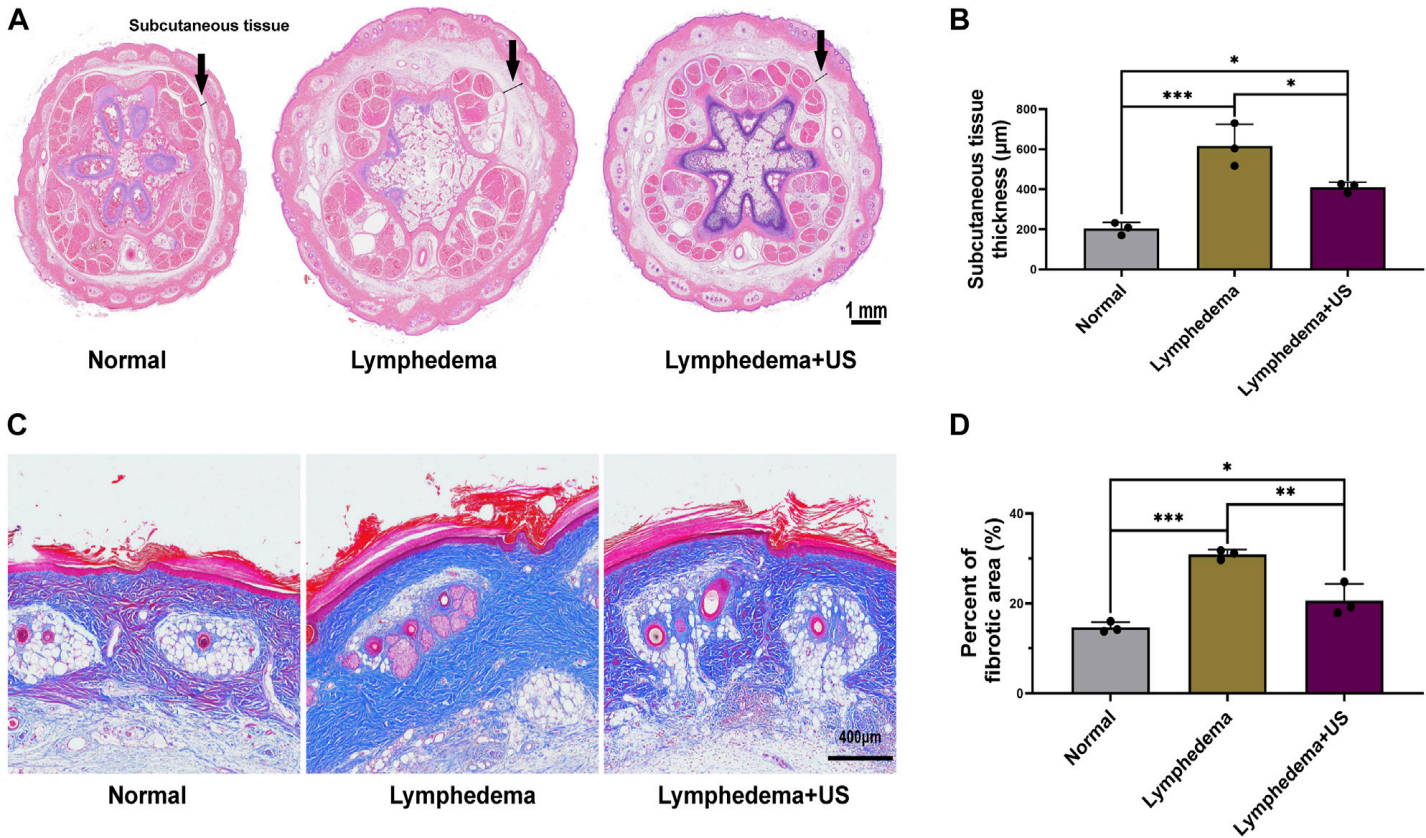
Histological photomicrographs of lymphedema site of the control and LIPUS groups. **(A,B)** HE staining showing the thickness of subcutaneous tissue at the lymphedema site, scale bar = 1 mm. **(C)** Masson’s staining to evaluate fibrotic tissue deposition revealed a decrease in fibrosis after LIPUS, scale bar = 400 μm. **(D)** Percent of the fibrotic area at the lymphedema site (*p* < 0.05, *n* = 3).

The rat tail volume (Figure 1D) was measured on days 0, 7, 14, 21, and 28. The tail volume of the ultrasonic treatment group and the non-treatment group exhibited significant differences after the operation (Figures 1B,C), with the largest difference (18%) on day 21 after the operation. The volume was measured by the drainage method. The histological analysis revealed that the thickness of subcutaneous tissue at the lymphedema site (Figure 2A) in the LIPUS treatment group decreased by 30% (HE staining) (Figure 2B) on day 28 after the operation of rat tail lymphatic vessels. The thickness of subcutaneous fibrous tissue decreased by 10% after ultrasonic treatment (Masson’s staining) (Figures 2C, D).

### 3.2 LIPUS reduced the lymphatic hyperplasia of rat tail lymphedema

**FIGURE 3:**
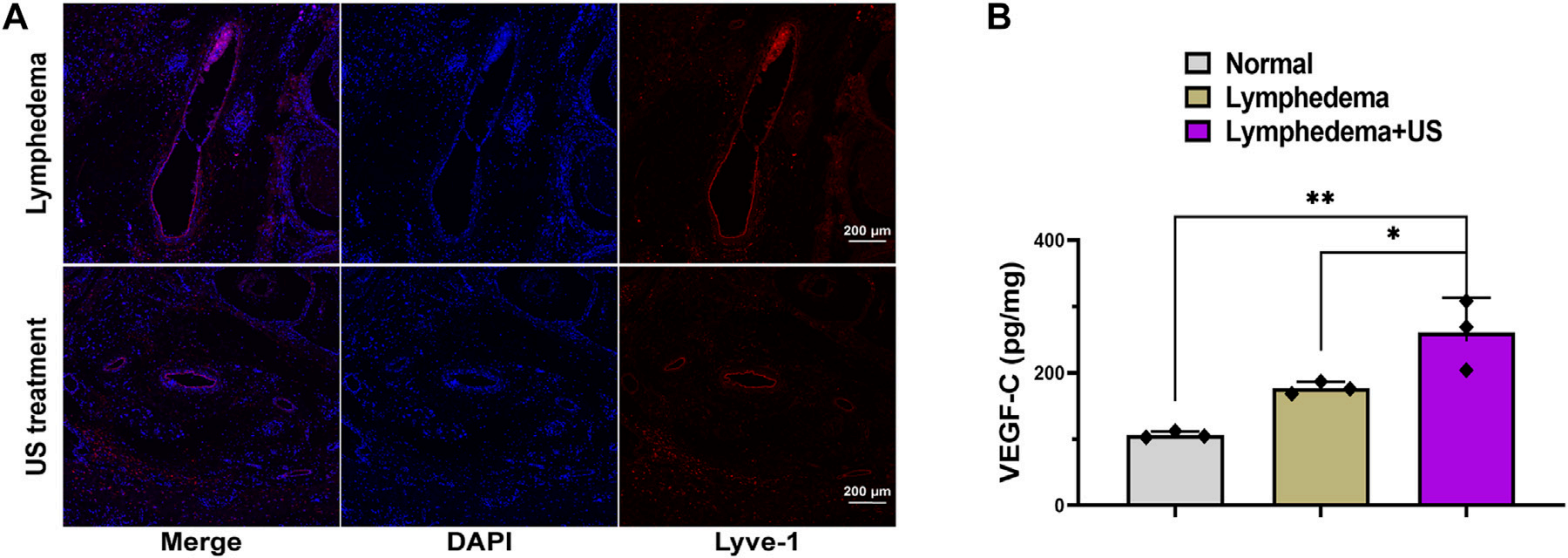
Lymphatic vessel in lymphedema tail after LIPUS treatment. **(A)** Immunofluorescence staining for LYVE-1 (red) showing lymphatic vessel 3 weeks after LIPUS, scale bar = 200 μm; LYVE-1: lymphatic vessel endothelial hyaluronan receptor 1. **(B)** The VEGF-C level at the lymphedema site. (*p* < 0.05, *n* = 3).

On day 28 after lymphatic vessel surgery, the skin and subcutaneous tissue were collected for homogenization, and the VEGF-C level of the LIPUS group was increased (Figure 3B), indicating that LIPUS treatment toadied in the growth of damaged lymphatic vessels.

The lyve-1 staining (Figure 3A) revealed lymphatic hyperplasia. The lymphatic vessels in the LIPUS treatment group exhibited a smaller cross-sectional area, whereas the lymphatic vessels of the lymphedema group exhibited a significantly expanded cross-sectional area and were swollen and thickened. The enhanced microcirculation might be the reason for the improved degree of lymphatic obstruction.

### 3.3 LIPUS reduced inflammation by regulating macrophage polarization

**FIGURE 4:**
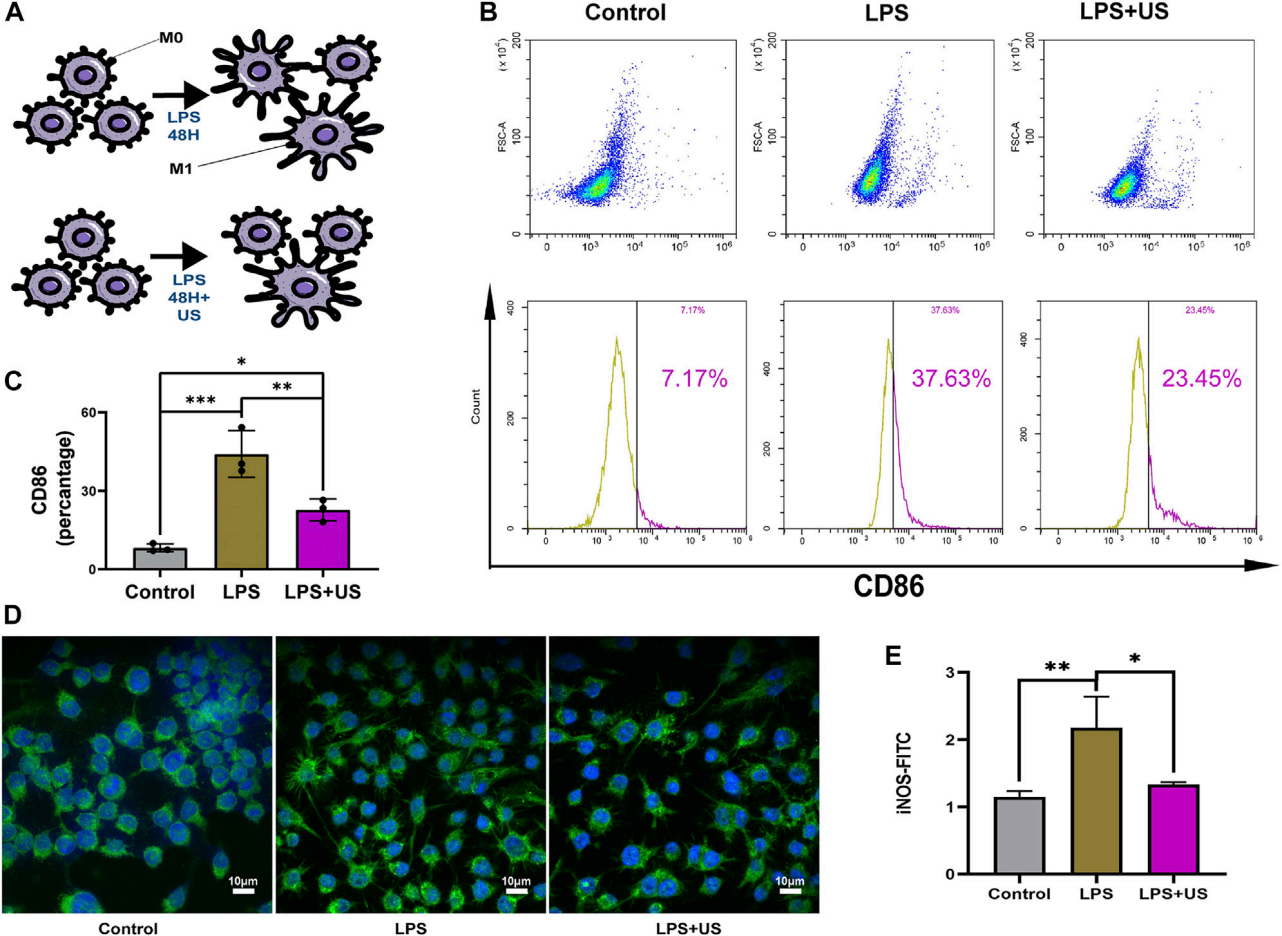
Cell experiments to examine the effect of LIPUS on macrophage polarization. **(A)** LIPUS regulates macrophage polarization. **(B,C)** The raw 264.7 cells were treated with LPS and LIPUS. Flow cytometry was performed to determine the percentage of CD86^+^ macrophages (*p* < 0.05, *n* = 3). **(D,E)** The intensity of fluorescence staining of iNOS.

A cellular model was used to study the cellular function of LIPUS. The raw264.7 cells were harvested using LPS (10 ng/mL), and then the expression of the phenotypic marker—CD86—associated with M1 macrophages was examined using flow cytometry. After the ultrasound treatment, the CD86 phenotype of LPS-treated raw264.7 cells decreased by 22% (Figure 4B).

iNOS was mainly expressed in M1 macrophage (Figure 4C). Compared with and without the LIPUS group, LIPUS reduced the iNOS fluorescence intensity (Figures 4D, E).

### 3.4 LIPUS promote microcirculation

**FIGURE 5:**
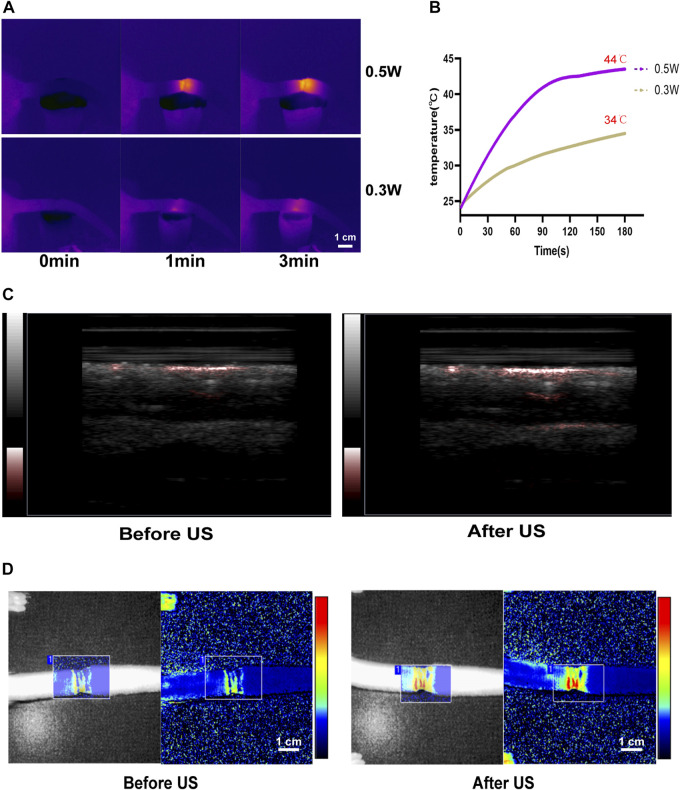
LIPUS enhances microcirculation in lymphedema tails. **(A,B)** The temperature of rat tail after LIPUS increase in different power density. **(C)** The blood flow of the rat tail increased after LIPUS treatment in the photoacoustic imaging system. **(D)** The blood flow of the rat tail increased after LIPUS treatment in the laser Doppler flowmetry (LDF).

After LIPUS treatment, the photoacoustic imaging system (Figure 5C) and the laser Doppler flowmetry (LDF) (Figure 5D) revealed an increase in the blood flow of the rat tail. Infrared temperature measurement was used to measure the temperature of the rat tail. The temperature increased after LIPUS but did not rise after reaching 44°C (Figures 5A, B). The skin and subcutaneous tissue could be heated to promote microcirculation.

## 4 Discussion

Ultrasonic examination—the most prevalent medical imaging modality worldwide (Wang et al., 2020)—is abundant, safe, portable, and inexpensive. In addition to rapidly expanding beyond traditional radiology and cardiology practices, Ultrasound also plays a unique role in treating various diseases. With such early visions, FRY et al. (1955) developed focused ultrasound, utilizing its ability to penetrate deeply into the human body and provide tight energy deposition in focused areas. Initial success with a therapeutic intervention was reported after its implementation. After years of significant progress, LIPUS was approved by the FDA for several diseases (Schandelmaier et al., 2017). Studies have demonstrated that LIPUS can reduce inflammation and accelerate vascular damage repair, and it has been widely used to treat various diseases (Harrison and Alt, 2021). Similarly, our results demonstrated that LIPUS could reduce secondary lymphedema by accelerating blood circulation and reducing inflammation.

We verified that LIPUS intervention significantly reduced the swelling in the rat tails model after lympectomy. The results demonstrated the effectiveness of LIPUS treatment in relieving lymphedema. However, the volume of the rat tail began to differ significantly after 21 days, indicating that LIPUS treatment requires a certain course of treatment. In the acute swelling period, emergency treatment may still be required, and LIPUS is more suitable for medium- and long-term treatment. Second, the difference between treatment groups diminished over time, possibly due to the self-limitation of the rat tail lymphedema model.

Guilherme et al. (Cuadrado et al., 2021) found that inflammation precedes fat deposition in lymphedema, indicating the importance of inflammatory macrophages in lymphedema. Studies have reported that LIPUS inhibits inflammatory responses by reducing the proportion of M1 macrophages. Ultrasound may affect the polarization of macrophages by inhibiting the production of pro-inflammatory cytokines such as interleukin-33 (IL-33), IL-6, IL-8, and IL-1β and suppressing intracellular signaling such as extracellular signal-regulated kinase (ERK) and MAPK (Zhang et al., 2020; Iacoponi et al., 2023). Similarly, our cellular experiments confirmed that LIPUS could alter the polarization of macrophages in response to inflammatory stimulation. Kusuyama et al. (2019) reported that LIPUS inhibited inflammatory cytokines such as IL-1 and TNF- α. This finding is consistent with our conclusion: repolarization may be one of the major causes of LIPUS that can reduce lymphedema inflammation.

In lymphedema, subcutaneous tissue thickening was the most significant pathological change. Moreover, fibrosis is crucial for pathological changes. Studies have demonstrated that fibrosis occurs not only in subcutaneous fat but also in lymph and neonatal lymphatic vessels. Fibrosis seriously affects the function of lymphatic vessels and aggravates lymphedema. Animal model studies of lymphedema demonstrated that many neonatal lymphatic vessels appeared in the lymphedema lesion 2 weeks after model creation, but the neonatal lymphatic vessel was irregular. The irregular lumens hindered the function of lymphatic function. After LIPUS intervention, the thickness of the subcutaneous tissue and fat layer of the tail decreased, tissue fibrosis diminished, and lymphatic function improved, demonstrating the effectiveness of LIPUS in relieving lymphedema.

Lymphatic obstruction leads to an increased degree of swelling in lymphedema. Patrick et al. (Mucka et al., 2016) found that reduced vascular permeability could hinder lymphatic drainage and aggravate lymphoedema swelling in Nrp2 deficient mice. In our study, the LIPUS treatment could promote microcirculation, improve vascular permeability, and increase blood flow; this is the short-term effect of ultrasound treatment (Kösters et al., 2017). Through mechanical stimulation, endogenous friction could promote blood and lymphatic circulation, improve the expression of VEGF-C, and accelerate the repair and lymphatic regeneration (Cheung et al., 2006). In the progression of lymphedema, lymphatic circulation disorder is the most important aggravating factor. We used LIPUS to stimulate the local circulatory ability, accelerate the lymphatic function of collateral compensation or stricture, and accelerate the repair function of the lymphatic system.

Our study revealed the relieving effect of LIPUS on secondary lymphedema. The results demonstrated that LIPUS could reduce lymphedema by regulating macrophage polarization and enhancing microcirculation. This finding offers a promising therapeutic way for lymphedema in the future. To overcome the existing ultrasonic treatment equipment, our team are working on a wearable ultrasound treatment device. It is also a practical attempt to improve the quality of life (QOL) of lymphedema patients.

## Data Availability

The original contributions presented in the study are included in the article/Supplementary Material, further inquiries can be directed to the corresponding authors.
