# Supplementary material for: Low-intensity pulsed ultrasound reduces lymphedema by regulating macrophage polarization and enhancing microcirculation
Source: Front Bioeng Biotechnol. 2023 May 5;11:1173169. doi: 10.3389/fbioe.2023.1173169 (PMC10198614; doi:10.3389/fbioe.2023.1173169)
Supplement: Supplementary file 1 [file DataSheet1.PDF]

Supplementary material

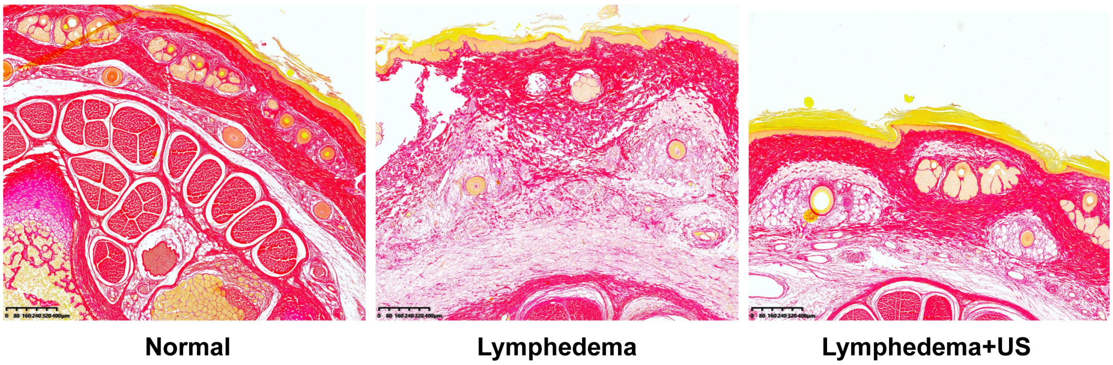

S1. Sirius staining for the lymphedema tissue, scale bar = 400 μm. The red stain indicates collagen fibrous tissue. LIPUS reduced swelling and fibrous(red) hyperplasia in lymphedema.

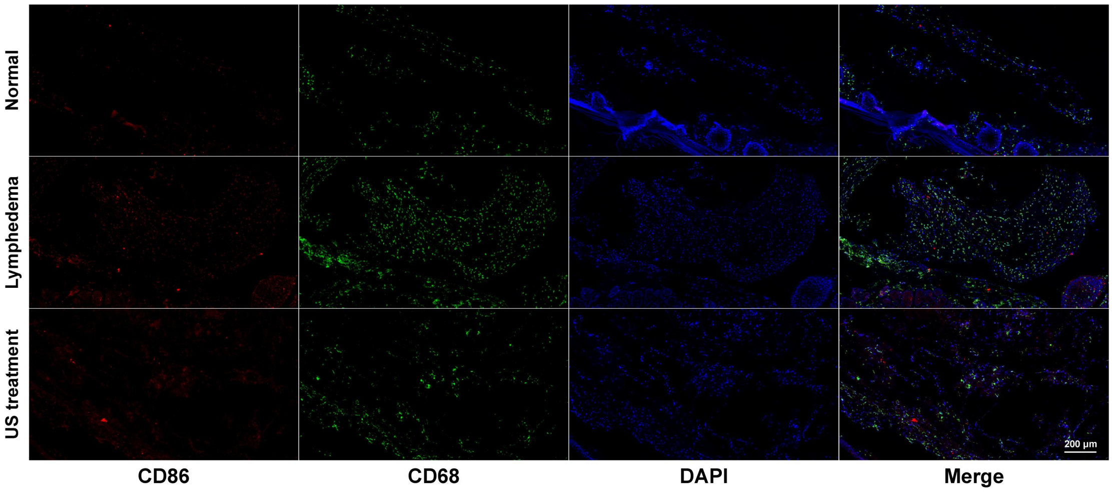

S2. Immunofluorescence staining for CD86(red) and CD68(green) showing macrophage polarization after LIPUS, scale bar = 200 μm; CD86: mainly express at M1 macrophages; CD86: express at all macrophages.

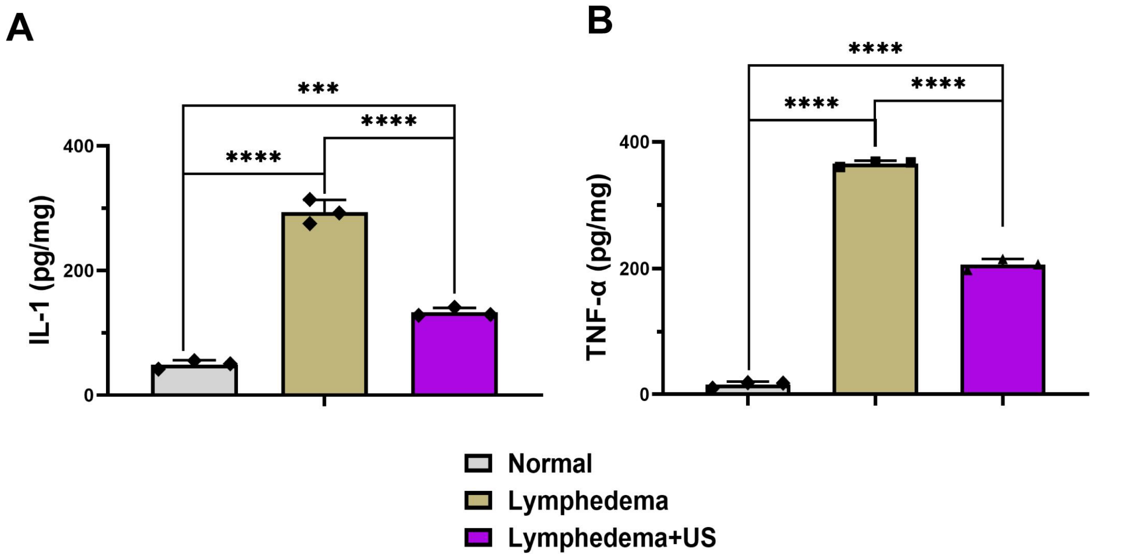

S3.(A) The IL-1 level at the lymphedema site. **(B)** The TNF-α level at the lymphedema site.($p < 0.05$, n = 3)
